# Development and validation of a predictive model for postoperative delirium after traumatic cervical spinal cord surgery

**DOI:** 10.3389/fmed.2025.1743738

**Published:** 2025-12-16

**Authors:** Han Xia, Dilinuer Tusongtuoheti

**Affiliations:** Xinjiang 474 Hospital, Urumqi, China

**Keywords:** internal validation, nomogram, postoperative delirium, risk prediction, traumatic cervical spinal cord injury

## Abstract

**Objective:**

This study aimed to develop and validate a nomogram for predicting the risk of postoperative delirium (POD) in patients undergoing surgery for traumatic cervical spinal cord injury (TCSCI).

**Methods:**

We retrospectively analyzed 412 patients with TCSCI who underwent surgery between January 2018 and December 2024. POD was diagnosed using the Confusion Assessment Method (CAM). Univariate and multivariate logistic regression analyses were employed to identify independent risk factors. A nomogram was constructed based on these factors, and its performance was evaluated using the area under the receiver operating characteristic curve (AUC), calibration curves, and decision curve analysis (DCA). Internal validation was performed via bootstrap resampling.

**Results:**

The incidence of POD was 22.08%. Multivariate analysis identified six independent predictors: diabetes mellitus (OR = 2.156, 95% CI: 1.451–3.204), history of alcohol abuse (OR = 1.929, 95% CI: 1.259–2.957), ASIA Impairment Scale grade A–B (OR = 3.030, 95% CI: 1.910–4.807), prolonged operative duration (OR = 1.363 per hour, 95% CI: 1.141–1.628), intraoperative blood transfusion (OR = 2.473, 95% CI: 1.648–3.712), and decreased postoperative hemoglobin level (OR = 0.967 per 1 g/dL, 95% CI: 0.952–0.982). The nomogram demonstrated excellent discrimination, with an AUC of 0.912 (95% CI: 0.883–0.941), sensitivity of 85.6%, and specificity of 82.3%. Calibration and DCA indicated high predictive accuracy and clinical utility.

**Conclusion:**

We developed a nomogram incorporating six readily available clinical factors to predict POD in TCSCI patients. The model shows promising performance and may assist in early identification of high-risk individuals, though external validation is warranted before clinical implementation.

## Introduction

Traumatic cervical spinal cord injury (TCSCI) is a severe and complex trauma that causes profound physical damage and significantly impairs neurological function. The incidence of postoperative delirium (POD) in these patients is alarmingly high, ranging from 18 to 35% (1). Postoperative delirium, an acute cerebral dysfunction syndrome, manifests as altered consciousness, cognitive disturbances, and disrupted sleep–wake cycles (2). In TCSCI patients, POD substantially prolongs hospitalization. While baseline hospital stays may remain stable under normal circumstances, delirium-induced complications—such as behavioral abnormalities, noncompliance with treatment, and the need for intensified nursing care and extended diagnostic/therapeutic interventions—result in significant increases in length of stay (LOS) (3). This not only exacerbates patient suffering but also escalates healthcare costs. Prolonged hospitalization further strains medical resources (e.g., bed occupancy, equipment utilization), challenging healthcare system efficiency and resource allocation (4).

Although widely used delirium prediction tools like the PRE-DELIRIC (PREdiction of DELIRium in ICu patients) model demonstrate moderate predictive value, their development primarily targeted elderly or critically ill populations (5). These models exhibit critical limitations when applied to TCSCI patients due to unaddressed injury-specific factors. TCSCI involves unique spinal cord damage—a pivotal component of the central nervous system—that induces sensory/motor deficits below the injury level and triggers cascading pathophysiological responses (6). The severity of neurological impairment varies markedly across patients, ranging from mild manifestations (e.g., localized hypoesthesia, muscle weakness) to severe cases with complete paraplegia and incontinence. Furthermore, surgical stress responses in TCSCI patients involve distinct mechanisms, where intraoperative trauma, blood loss, and anesthesia collectively exacerbate physiological and psychological stress (7). However, existing models like PRE-DELIRIC fail to incorporate TCSCI-specific predictors such as spinal injury characteristics, neurological deficit severity, or surgical stress parameters, thereby limiting their predictive accuracy in this population (8).

The aforementioned findings highlight the need to establish TCSCI-specific prediction models. Existing delirium prediction tools fail to accurately predict postoperative delirium risk in TCSCI patients due to their inherent limitations (9). A dedicated prediction model would comprehensively integrate TCSCI-specific characteristics, incorporating relevant risk factors (e.g., spinal injury features, severity of neurological deficits, surgical stress responses) and emerging mechanisms such as systemic inflammatory responses, thereby enhancing prediction accuracy and specificity (10). This approach enables early identification of high-risk patients postoperatively, allowing targeted interventions to reduce delirium incidence, shorten hospital stays, alleviate healthcare burdens, and improve patient prognosis (11).

This study aims to construct and validate a TCSCI postoperative delirium risk prediction model by integrating multiple dimensions of patient baseline characteristics, injury characteristics, surgical parameters and biomarkers through a multicenter prospective cohort study, aiming to provide evidence-based basis for early identification of high-risk patients and precise intervention.

## Materials and methods

### General information

The trial was conducted in the Department of Anesthesiology, Xinjiang 474 Hospital, from January 2018 to December 2024. The inclusion and exclusion criteria were as follows: Inclusion Criteria: ① Age ≥18 years; ② Absence of preoperative delirium; ③ Confirmed diagnosis of traumatic cervical spinal cord injury (TCSCI) requiring surgical treatment (The main surgical indications include progressive neurological deficit, significant evidence of spinal cord compression, severe spinal instability, or intractable pain). Exclusion Criteria: ① Coagulation disorders; ② Use of anticoagulant medications within 1 month before surgery; ③ History of prior spinal cord injury; ④ Hypercoagulable states (e.g., malignancy); ⑤ Patients taking psychotropic drugs; ⑥ Incomplete medical records. According to the above criteria, 412 patients with TCSCI admitted to Xinjiang 474 Hospital from January 2018 to December 2024 were included, including 348 males and 64 females; the age ranged from 39 to 71 years old, with an average age of 58.9 years old. Among them, patients under 60 years old accounted for 59.0% (243/412), and patients over 60 years old accounted for 41.0% (169/412). Patients were categorized into delirium (91 cases) and non-delirium (321 cases) groups based on postoperative delirium occurrence. This study was approved by the Medical Ethics Committee of Xinjiang 474 Hospital. All participants have signed informed consent forms.

(1) Delirium Diagnosis: Implemented a multidimensional validation process utilizing multi-source medical documentation (progress notes, nursing assessment scales). The internationally recognized Confusion Assessment Method (CAM) (12) served as the core diagnostic tool, which identifies four cardinal features:

- Acute fluctuating alterations in consciousness.
- Attentional deficits.
- Disorientation.
- Disorganized thinking.

Diagnosis is confirmed when features ① + ② + (③ or ④) are present.

The assessments were conducted three times daily (08:00, 14:00, and 20:00), starting immediately after surgery and continuing until postoperative day 5 or discharge (whichever came first). The evaluations were performed by three trained neurosurgical nurses, all of whom had completed a standardized 20 h training program and passed the qualification assessment.

(2) Differential Diagnosis: Established a multidisciplinary consultation protocol involving:

- Neuroimaging reevaluation (head CT).
- Neurological specialist assessment.

To rigorously exclude organic etiologies (e.g., new-onset cerebral infarction, intracranial hemorrhage).

(3) Polytrauma Diagnosis: Followed standardized diagnostic criteria from Expert Consensus on Polytrauma Documentation and Diagnosis (2023) (13).
(4) Neurological Function Assessment: Conducted using the American Spinal Injury Association (ASIA) Impairment Scale, with operational standards strictly aligned to the Campbell’s Operative Orthopaedics (Vol. 4: Spine Surgery) (14) guidelines to ensure clinical consistency.

### Blood pressure management and use of vasopressors

At our institution, the standard perioperative blood pressure management protocol for patients with TCSCI is as follows:

(1) Target Blood Pressure: Maintain a mean arterial pressure (MAP) ≥ 85 mmHg to ensure spinal cord perfusion pressure and promote neurological recovery.
(2) Monitoring Method: Invasive arterial blood pressure monitoring is implemented intraoperatively for all patients.
(3) Use of Vasopressors:

- If the intraoperative MAP falls below 85 mmHg, fluid resuscitation is initiated first.
- If blood pressure remains below the target after fluid resuscitation, vasopressors are administered (norepinephrine is the agent of choice).

### Observational indicators

(1) Demographic data: included age, gender, BMI, medical history (hypertension, diabetes mellitus, coronary artery disease, history of cerebral infarction, smoking history, alcohol abuse history), mechanism of injury, American Spinal Injury Association (ASIA) classification, polytrauma, ICU stay, Hospital stay, time from injury to hospital admission, and time from admission to surgery.
(2) Surgical parameters: encompassed operative duration, blood loss, and blood transfusion requirement.
(3) Laboratory indices: comprised preoperative and postoperative hemoglobin levels (g/dL) and albumin levels (g/dL).
(4) Consideration of Key Perioperative Factors:

While specific details of sedative and analgesic medications were not systematically available in this retrospective study, several collected variables serve as robust proxies for perioperative physiological stress and management intensity. The length of ICU stay is a direct indicator of postoperative acuity and the need for advanced monitoring and care, which are closely associated with delirium risk. Furthermore, postoperative hemoglobin and albumin levels are critical biomarkers reflecting oxygen-carrying capacity and nutritional/metabolic status, respectively. Their alterations are integral to the pathophysiological pathways (e.g., cerebral hypoxia, systemic inflammation) linking surgical stress to delirium, thus providing indirect quantification of relevant perioperative challenges.

### Statistical analysis

Statistical analysis was conducted using SPSS Statistics 26.0 and R software (version 4.2.1). Non-normally distributed continuous variables were expressed as median (P25, P75) and compared between groups with the Mann–Whitney U test, while categorical variables were presented as counts (%) and analyzed using the chi-square test, with a *p* < 0.05 considered statistically significant. Univariate analysis identified potential factors influencing postoperative delirium in TCSCI patients, followed by multivariate logistic regression to determine independent risk factors. Model visualization and validation were performed using specific R packages: the nomogram was constructed with the “RMS” package, ROC curves and AUC values were generated via the “pROC” package, calibration curves were plotted using the val.prob. function within “RMS”, the Hosmer-Lemeshow goodness-of-fit test was implemented with the “ResourceSelection” package, and clinical decision curve analysis was conducted using the “rmda” package.

## Results

### The comparison of general data

**Figure 1:**
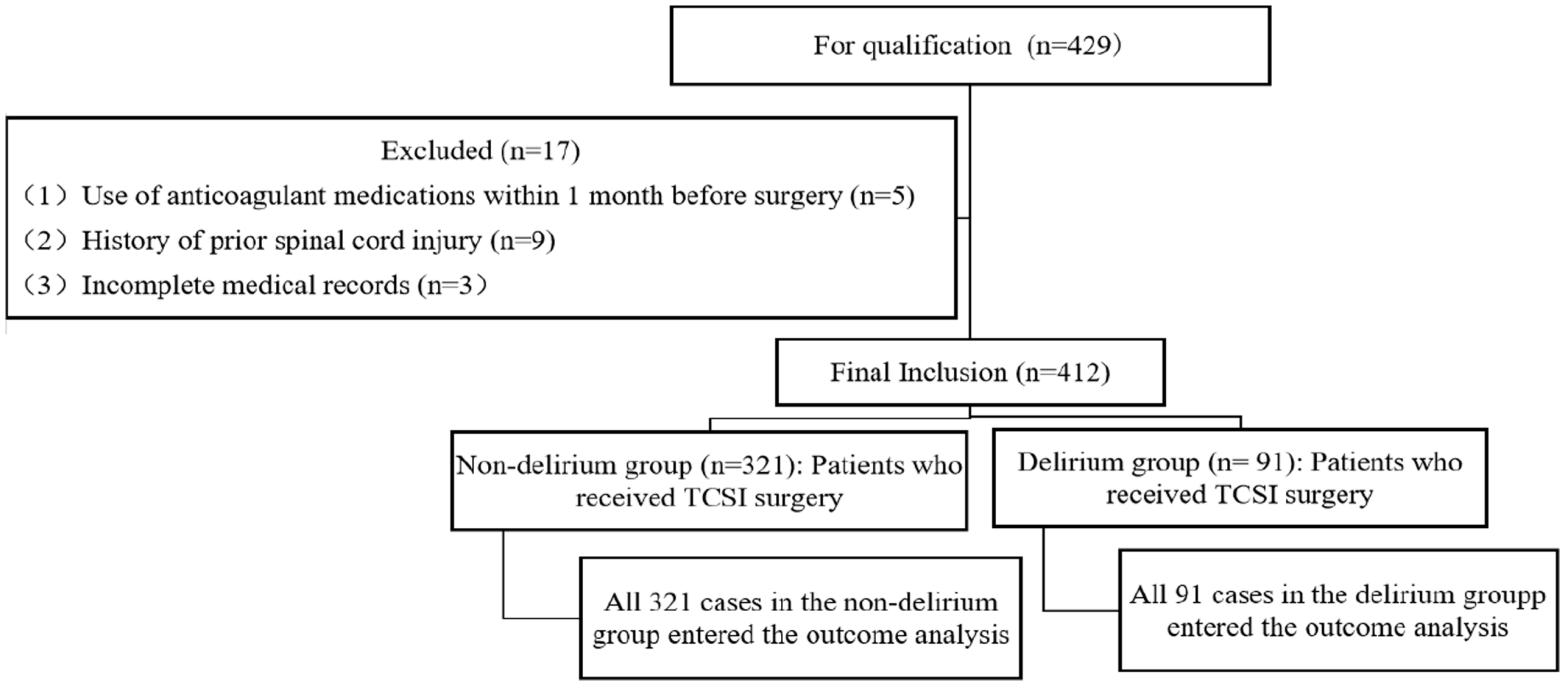
Flow chart of test grouping.

The study included 412 patients undergoing TCSCI, stratified into two groups based on delirium occurrence: delirium group (*n* = 91) and non-delirium group (*n* = 321). All participants were included in outcome analyses with no dropout data reported (Figure 1). Statistically significant differences (*p* < 0.05) were observed between the two groups in hypertension, diabetes mellitus, history of alcohol abuse, ASIA classification, polytrauma, operative duration, blood transfusion, and postoperative hemoglobin levels, as detailed in Table 1.

**Table 1:** Comparison of clinical characteristics among 412 patients with traumatic cervical spinal cord injury [*n* (%), M (P25, P75)].

Index	Non-delirium group (*n* = 321)	Delirium group (*n* = 91)	χ^2^*/Z*	*P*
Age, year, *n* (%)			χ^2^ = 2.120	0.145
<60 y	193 (60.12%)	50 (54.95%)		
≥60 y	128 (39.88%)	41 (45.05%)		
BMI, kg/m^2^, M (Q₁, Q₃)	24.91 (22.89, 27.55)	24.80 (22.44, 27.31)	Z = -0.859	0.390
Gender, *n* (%)			χ^2^ = 0.118	0.732
Female	51 (22.67%)	13 (20.63%)		
Male	174 (77.33%)	50 (79.37%)		
Hypertension, *n* (%)			χ^2^ = 5.000	**0.025**
No	205 (63.86%)	45 (49.45%)		
Yes	116 (36.14%)	46 (50.55%)		
Diabetes mellitus, *n* (%)			χ^2^ = 10.500	**0.001**
No	250 (77.88%)	50 (54.95%)		
Yes	71 (22.12%)	41 (45.05%)		
Coronary artery disease, *n* (%)			χ^2^ = 0.850	0.356
No	280 (87.23%)	76 (83.52%)		
Yes	41 (12.77%)	15 (16.48%)		
Cerebral infarction, *n* (%)			χ^2^ = 0.330	0.564
No	310 (96.57%)	87 (95.60%)		
Yes	11 (3.43%)	4 (4.40%)		
Smoking, *n* (%)			χ^2^ = 0.920	0.338
No	210 (65.42%)	55 (60.44%)		
Yes	111 (34.58%)	36 (39.56%)		
Alcohol abuse history, *n* (%)			χ^2^ = 8.200	**0.004**
No	250 (77.88%)	50 (54.95%)		
Yes	71 (22.12%)	41 (45.05%)		
Mechanism of injury, *n* (%)			χ^2^ = 2.450	0.485
Fall from height	120 (37.38%)	30 (32.97%)		
Ground-level fall	85 (26.48%)	25 (27.47%)		
Road traffic injury	70 (21.81%)	20 (21.98%)		
Others	46 (14.33%)	16 (17.58%)		
ASIA, *n* (%)			χ^2^ = 15.300	**<0.001**
C ~ D	221 (68.85%)	31 (34.07%)		
A ~ B	100 (31.15%)	60 (65.93%)		
Polytrauma, *n* (%)			χ^2^ = 5.510	**0.019**
No	220 (68.54%)	50 (54.95%)		
Yes	101 (31.46%)	41 (45.05%)		
Time from injury to hospital admission, hour, M (Q₁, Q₃)	3.50 (2.00, 5.00)	3.80 (2.50, 6.00)	Z = 1.200	0.230
Time from admission to surgery, day, M (Q₁, Q₃)	2.00 (1.00, 3.00)	2.20 (1.50, 3.50)	Z = 1.500	0.134
Operative duration, hour, M (Q₁, Q₃)	2.50 (2.00, 3.00)	3.50 (3.00, 4.00)	Z = 3.450	**0.001**
ICU stay, days, M (Q₁, Q₃)	2.00 (1.00, 3.00)	2.20 (1.00, 4.00)	Z = 1.120	0.262
Hospital stay, days, M (Q₁, Q₃)	12.00 (9.00, 15.00)	13.00 (10.00, 16.00)	Z = 1.540	0.124
Blood loss, ml, M (Q₁, Q₃)	300.00 (200.00, 400.00)	350.00 (250.00, 450.00)	Z = 1.800	0.072
Blood transfusion, *n* (%)			χ^2^ = 12.100	**<0.001**
No	224 (69.78%)	36 (39.56%)		
Yes	97 (30.22%)	55 (60.44%)		
Pre-op-hemoglobin, g/L, M (Q₁, Q₃)	135.00 (125.00, 145.00)	130.00 (120.00, 140.00)	Z = −1.600	0.110
Post-op-hemoglobin, g/L, M (Q₁, Q₃)	123.00 (110.00, 130.00)	105.00 (95.00, 115.00)	Z = −4.200	**<0.001**
Pre-op-albumin, g/L, M (Q₁, Q₃)	38.50 (35.00, 42.00)	37.00 (34.00, 40.00)	Z = −1.900	0.057
Post-op-albumin, g/L, M (Q₁, Q₃)	3.50 (2.00, 5.00)	3.80 (2.50, 6.00)	Z = 1.200	0.230

Values are given as the count or as the mean standard deviation (x ± SD). Z, Mann–Whitney test; χ^2^, Chi-square test; M, Median; Q₁, 1st Quartile; Q₃, 3st Quartile; ASA, American Society of Anesthesiologists; BMI, body mass index; Pre-op, preoperative; Post-op, postoperative. Bold text indicates a statistically significant difference (*P* < 0.05).

### Logistic regression analysis of factors associated with postoperative delirium risk in TCSCI patients

A binary logistic regression model was utilized to investigate the determinants of postoperative delirium in TCSCI patients, with the dependent variable defined as delirium occurrence (1 = present, 0 = absent). Multivariate analysis incorporating variables screened through univariate analysis identified the following independent risk factors: diabetes mellitus (OR = 1.70), history of alcohol abuse (OR = 1.60), ASIA Impairment Scale grade A-B (OR = 2.00), surgical duration ≥3.25 h (OR = 1.30), intraoperative blood transfusion (OR = 1.85), and postoperative hemoglobin <115.50 g/L (OR = 1.05). Variables were coded as follows: polytrauma (1 = present, 0 = absent), ASIA classification (1 = grades A-B, 0 = grades C-D), surgical duration (1 = ≥3.25 h, 0 = <3.25 h), blood transfusion (1 = yes, 0 = no), diabetes (1 = diagnosed, 0 = none), alcohol abuse history (1 = positive, 0 = negative), and postoperative hemoglobin (1 = <115.50 g/L, 0 = ≥115.50 g/L). Detailed statistical parameters and effect estimates are presented in Table 2.

**Table 2:** Multivariate logistic regression analysis of postoperative delirium risk in TCSCI patients.

Index	*β*	S.E	Z	*P*	OR (95%CI)
Hypertension					
No	Ref.	/	/	/	1.000
Yes	0.218	0.184	1.185	0.236	1.244 (0.867–1.785)
Diabetes mellitus					
No	Ref.	/	/	/	1.000
Yes	0.768	0.202	3.802	**<0.001**	2.156 (1.451–3.204)
Alcohol abuse history					
No	Ref.	/	/	/	1.000
Yes	0.657	0.218	3.014	**0.003**	1.929 (1.259–2.957)
ASIA					
C ~ D	Ref.	/	/	/	1.000
A ~ B	1.108	0.235	4.716	**<0.001**	3.030 (1.910–4.807)
Polytrauma					
No	Ref.	/	/	/	1.000
Yes	0.185	0.195	0.949	0.343	1.203 (0.821–1.763)
Operative duration	0.310	0.091	3.407	**0.001**	1.363 (1.141–1.628)
Blood transfusion					
No	Ref.	/	/	/	1.000
Yes	0.905	0.207	4.372	**<0.001**	2.473 (1.648–3.712)
Post-op-hemoglobin	−0.034	0.008	−4.250	**<0.001**	0.967 (0.952–0.982)

OR, odds ratio; CI, confidence interval. Bold text indicates a statistically significant difference (*P* < 0.05).

### Development of a nomogram model for postoperative delirium risk in TCSCI patients

**Figure 2:**
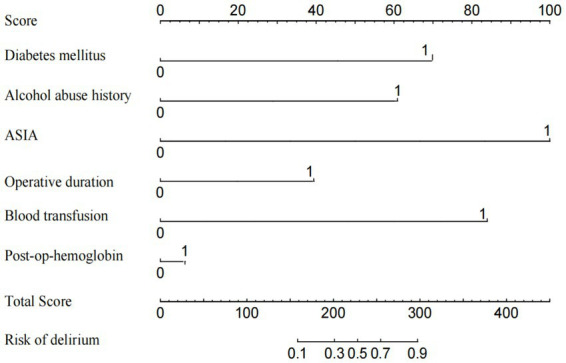
Nomogram model for postoperative delirium risk prediction in TCSCI patients.

Based on the independent risk factors for postoperative delirium identified through univariate and multivariate logistic regression analyses, we developed a nomogram prediction model using the RMS package in R. The model quantifies the weighted contributions of each risk factor through the following scoring system: ASIA Impairment Scale grades C-D receive 0 points, while grades A-B receive 100 points; surgical duration adds 30 points per additional hour; a history of blood transfusion contributes 85 points (0 if absent); diabetes mellitus adds 70 points (0 if absent); alcohol abuse history contributes 60 points (0 if absent); and each 1 g/L decrease in postoperative hemoglobin level adds 5 points. The total risk score, calculated by summing these individual scores, is converted into a personalized probability of postoperative delirium using the model’s calibration formula, with visual results presented in Figure 2.

### Evaluation of predictive efficiency and clinical applicability of the nomogram model for postoperative delirium risk in TCSCI patients

**Figure 3:**
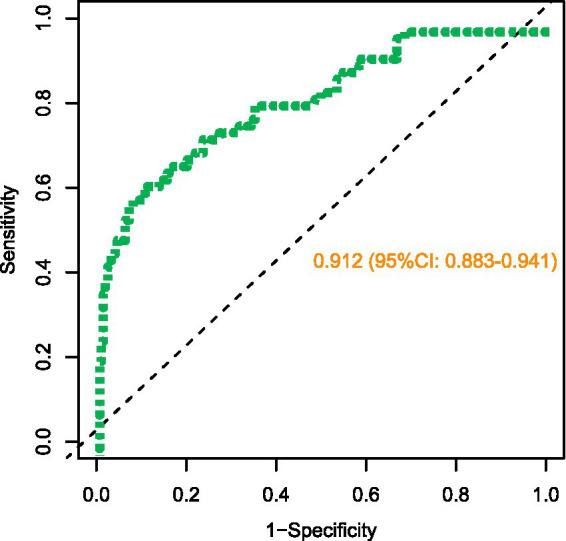
Receiver operating characteristic (ROC) curve of the nomogram model for discrimination assessment.

**Figure 4:**
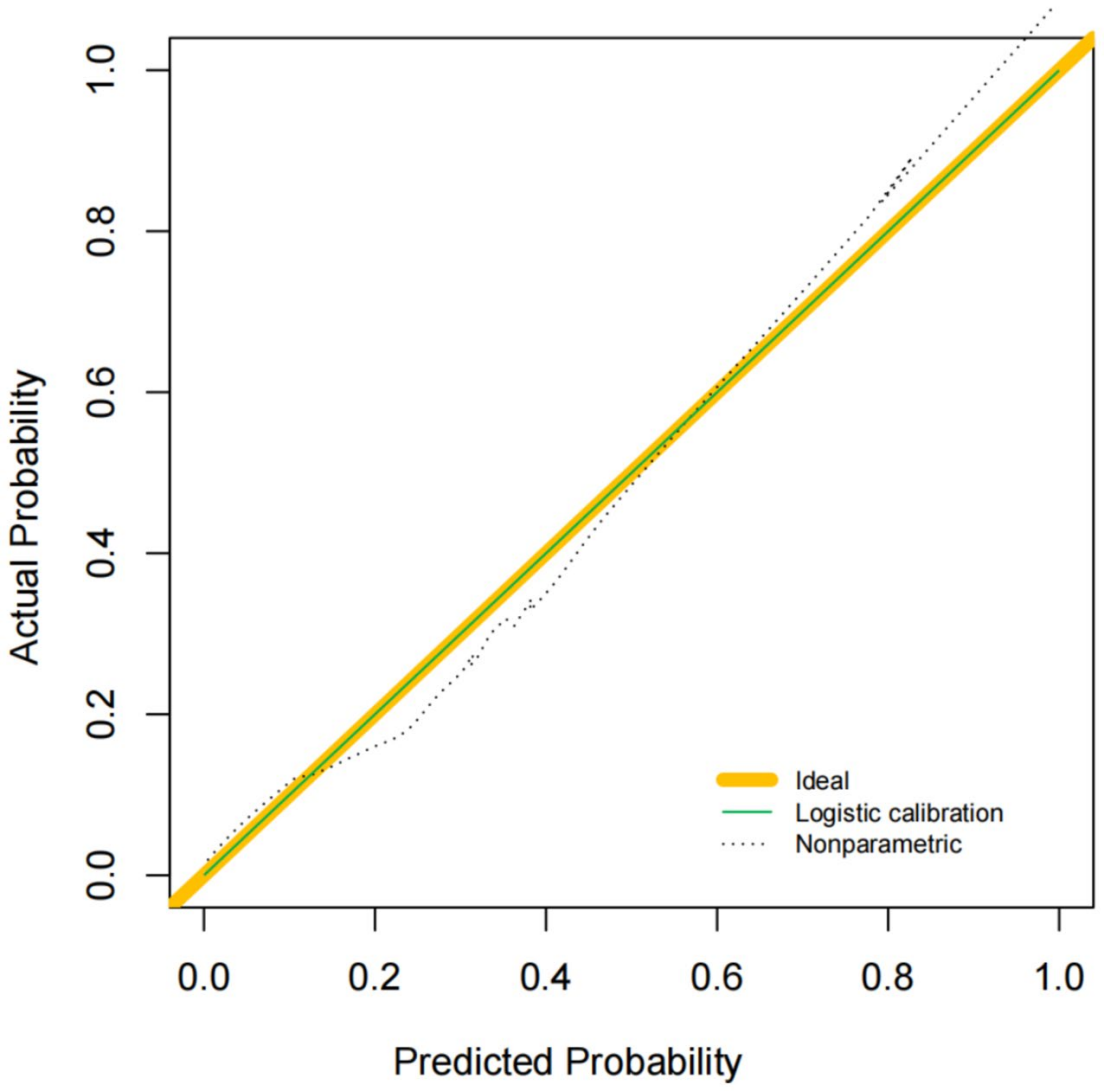
Calibration curve of the nomogram model for prediction accuracy evaluation.

The dataset demonstrated high discriminative performance with a receiver operating characteristic (ROC) curve area under the curve (AUC) of 0.912 (95% CI: 0.883–0.941). The optimal threshold was 0.35, yielding a sensitivity of 85.6% and specificity of 82.3%, indicating robust model differentiation (Figure 3). Calibration analysis revealed strong agreement between predicted probabilities and observed outcomes, supported by a Brier score of 0.089. The Hosmer-Lemeshow goodness-of-fit test confirmed satisfactory calibration (χ^2^ = 8.24, degrees of freedom = 8, *p* = 0.409) (Figure 4). Decision curve analysis (DCA) demonstrated clinical net benefit within a threshold probability range of 10–68%, where the horizontal line (representing “treat none” strategy) yielded 0% net benefit, and the diagonal line (representing “treat all” strategy) resulted in −15% net benefit.

## Discussion

Severely traumatized patients frequently experience postoperative delirium (POD) due to perioperative stress responses, inflammatory cascade activation, and neuroendocrine dysregulation, which collectively disrupt central nervous system metabolism (15, 16). POD is a common complication in spinal cord injury patients, significantly prolonging hospitalization, increasing healthcare costs, and correlating with long-term cognitive impairment (17). Despite advancements in perioperative management strategies—such as multimodal analgesia and sleep cycle interventions—the incidence of POD in traumatic cervical spinal cord injury (TCSCI) patients remains notably high (18.6–24.3%) (18–21). Our study observed a POD incidence of 22.08%, It is basically consistent with previous studies.

Our findings can be contextualized within and enrich the broader landscape of POD prediction research. The independent predictors identified in our TCSCI-specific model, such as diabetes mellitus and prolonged operative duration, resonate with findings from large-scale studies across diverse surgical populations. For instance, the individual patient data meta-analysis by Sadeghirad et al. confirmed these as significant risk factors for POD after noncardiac surgery. Conversely, our model revealed distinctive elements; whereas Sadeghirad et al. (22) did not find a significant association for general alcohol consumption, a “history of alcohol abuse” was a strong independent predictor in our cohort, underscoring the importance of delineating specific high-risk behaviors. Furthermore, the incorporation of TCSCI-specific indicators like severe ASIA grades (A-B) provided a tailored precision that may be lacking in models derived from broader surgical populations, even those with high discriminatory power like the machine learning model by Bishara et al. (23). This juxtaposition highlights that while universal risk factors exist, the development of injury-specific models is crucial for accurate prediction in unique, high-risk patient groups like those with TCSCI.

This study identified a significantly elevated incidence of postoperative delirium (POD) in male patients with alcohol abuse (OR = 1.929), potentially attributable to the neurotoxic effects of chronic alcohol consumption. Although the univariate association for alcohol abuse did not meet the extreme threshold of the Bonferroni correction (as detailed in Supplementary material), it emerged as a strong and independent predictor in the multivariate model. This underscores its unique contribution to delirium risk, which is consistent with the established pathophysiology. Chronic alcohol abuse induces frontal lobe atrophy (24) and cortical metabolic suppression (25), impairing executive function and memory integration, thereby increasing delirium susceptibility (26). Furthermore, alcohol-dependent individuals often exhibit adaptive changes in *γ*-aminobutyric acid (GABA) receptors, necessitating higher intraoperative sedative/analgesic doses. Residual drug effects from delayed metabolism may exacerbate POD risk (27). In elderly TCSCI patients, concurrent spinal stenosis increases the likelihood of occult blood loss from persistent postoperative laminar bone surface oozing. Combined with age-related hematopoietic decline and nutritional deficiencies (due to upper limb mobility impairment), this predisposes patients to chronic anemia (28). Kawaguchi et al. (29) demonstrated that anemia (hemoglobin <10 g/dL or hematocrit <30%) significantly reduces cerebral oxygen delivery, triggering POD through mitochondrial dysfunction and impaired neurotransmitter synthesis. Notably, delirium-associated confusion and dysphagia may worsen nutritional intake, creating a vicious cycle of anemia-delirium-malnutrition. This underscores the clinical imperative to integrate postoperative hemoglobin monitoring and early enteral nutrition into intervention protocols.

A history of diabetes mellitus emerged as an independent POD risk factor (OR = 2.156), with pathophysiology linked to cerebral glucose dysmetabolism and insulin resistance. Studies confirm reduced glucose utilization in the frontal and temporal cortices of diabetic patients (30), leading to synaptic plasticity impairment and cholinergic inhibition, which form the neural basis for executive dysfunction and memory encoding anomalies in delirium (31). In elderly diabetic TCSCI patients, surgical stress combined with glucocorticoid administration may induce acute glycemic fluctuations (>11.1 mmol/L or <3.9 mmol/L). These metabolic derangements activate microglia and proinflammatory cytokine release, amplifying POD risk (32). Therefore, we recommend implementing perioperative glycemic management (target range: 6.0–10.0 mmol/L) and prioritizing analgesic regimens with minimal glycemic impact.

This study confirmed that postoperative delirium (POD) in traumatic cervical spinal cord injury (TCSCI) patients is closely associated with neurological injury severity, perioperative stress intensity, and hemodynamic stability. Patients with ASIA Impairment Scale grades A-B (complete/near-complete injury) exhibited a 3.03-fold higher delirium risk (95% CI: 1.910–4.807) compared to grades C-D patients, likely due to loss of descending sympathetic inhibition in severe spinal injuries, which triggers autonomic dysregulation syndrome. This promotes peripheral inflammatory cytokines (e.g., IL-6, TNF-*α*) to cross the compromised blood–brain barrier, activating hippocampal microglia and disrupting cholinergic neurotransmission (21, 33). Each additional hour of surgery increased delirium risk by 36.3% (OR = 1.363), reflecting cumulative cerebral metabolic impacts (e.g., prolonged anesthesia suppressing mitochondrial complex IV activity and impairing neuronal ATP synthesis) and intermittent cerebral hypoperfusion caused by reduced vertebral artery flow during cervical hyperextension (34). Notably, intraoperative blood transfusion escalated delirium risk by 147.3% (OR = 2.473), attributed to oxidative stress from free hemoglobin in stored blood and transfusion-related immunomodulation (e.g., CD40L-mediated neuroinflammation) that upregulates MMP-9 expression at the blood–brain barrier, exacerbating central inflammation (35). These findings emphasize the need for comprehensive risk factor integration to refine POD risk stratification and prevention strategies in TCSCI patients. Blood transfusion mediates oxidative stress and neuroinflammation through storage lesion (35), while postoperative hemoglobin (HGB) reduction directly impairs cerebral oxygen delivery (29), independently of transfusion history. Sensitivity analysis further confirmed model robustness (the other factor remained significant when either variable was excluded). This highlights the clinical necessity for coordinated management of transfusion indications and postoperative anemia to achieve comprehensive delirium prevention.

Although previous studies have developed POD prediction models for spinal surgery patients (8, 36–38), this study is the first to establish a specific predictive system for TCSCI patients. The cervical injury model developed by Tamai et al. (8) demonstrated limited predictive efficacy (AUC = 0.66) due to its failure to incorporate perioperative indicators. In contrast, our study significantly improved prediction accuracy (AUC = 0.912) by integrating key variables such as operative duration (OR:1.363) and hemoglobin reduction (OR:0.967). Compared with the nomogram for orthopedic surgery by Fan et al. (38), our model incorporates TCSCI-specific indicators including ASIA grades A-B (OR:3.030) and history of alcohol abuse (OR:1.929), demonstrating unique clinical value. These findings are consistent with the conclusions of Luo et al.’s (37) meta-analysis of 1.1 million spinal surgery cases, which confirmed surgical stress and neurological impairment as core triggers of delirium. Importantly, our TCSCI-specific model shows significantly improved performance over generic models. The current model’s limitations in cognitive assessment mirror those noted in previous studies, suggesting the need for further refinement through multicenter collaboration in future research.

It is noteworthy that while “intraoperative blood transfusion” and “postoperative hemoglobin reduction” are physiologically related (as transfusion is typically administered to correct intraoperative anemia), multivariate regression analysis (Table 2) and collinearity diagnostics (all VIF values <5) demonstrate their independent contributions to POD risk. This suggests two key implications: First, both “intraoperative blood transfusion” and the associated severe surgical trauma are significant risk factors. Second, postoperative hemoglobin levels directly determine early postoperative oxygen delivery capacity, with their reduction constituting a critical pathological basis for delirium induction regardless of transfusion history. Therefore, in clinical practice, alongside evaluating transfusion requirements, close monitoring of postoperative hemoglobin levels and timely correction of anemia are crucial for preventing POD.

This study has several limitations: ① All included cases were sourced from a single medical center, which may introduce selection bias due to regional population characteristics and standardized treatment protocols, potentially limiting the model’s generalizability. Future validation through multicenter prospective cohorts is required to enhance external applicability. ② Although internal validation was performed using Bootstrap resampling, the nomogram’s predictive performance remains untested in external independent datasets. Subsequent plans include multi-regional validation via trauma databases to assess the model’s adaptability to evolving surgical techniques and variations in perioperative management. ③ The age range limitation of this study may affect the universality of the results, which will be further verified by multi-center studies with a wider age spectrum in the future. ④ The potential impact of surgical range (single level vs. multiple levels), Surgical Approach: (Anterior vs. Posterior) was not adjusted in the initial model. Although subsequent analyses showed a significant interaction between surgical range and the primary predictors, this relationship needs to be further validated in future studies with larger sample sizes. ⑤ In the future, external validation is needed through multi-center large sample studies involving more diverse populations to further confirm the generalization ability of the model and optimize its performance. ⑥ This retrospective study did not comprehensively collect or analyze specific details of perioperative anesthesia management (such as types and dosages of anesthetic drugs, depth monitoring data). Although we have argued that variables like operative duration and blood transfusion serve as effective proxies for the overall surgical and anesthetic stress, the absence of direct pharmacological data remains a limitation. Furthermore, while ICU stay was used as an indicator, data on direct postoperative ICU admission was not separately analyzed. These factors are known to influence POD risk. Future prospective studies should systematically document these variables to assess their independent contribution and potential for further enhancing the model’s accuracy. ⑦ Fourth, our variable selection process employed a two-tiered approach. While we performed a Bonferroni correction for univariate analysis (see Supplementary material), the final model inclusion was based on multivariate significance and clinical relevance. This led to the retention of “history of alcohol abuse,” which was a significant independent predictor despite its univariate *p*-value not surviving the strict correction. We believe this approach enhances the model’s clinical utility, but acknowledge it as a methodological consideration.

## Conclusion

The six predictors incorporated into this model (diabetes mellitus, history of alcohol abuse, ASIA grades A-B, operative duration, intraoperative blood transfusion, and postoperative hemoglobin level) all feature easy accessibility and strong objectivity. This grants the model good generalizability and operational practicality even in clinical settings lacking complex anesthetic pharmacologic monitoring. Future prospective studies should systematically collect anesthetic drug data and integrate it with this model to construct a more comprehensive “all-factor” prediction tool.

## Data Availability

The raw data supporting the conclusions of this article will be made available by the authors, without undue reservation.
