# Supplementary material for: Development and validation of a predictive model for postoperative delirium after traumatic cervical spinal cord surgery
Source: Front Med (Lausanne). 2025 Dec 16;12:1743738. doi: 10.3389/fmed.2025.1743738 (PMC12715597; doi:10.3389/fmed.2025.1743738)
Supplement: Supplementary file 1 [file Table_1.DOCX]

***Table1: Re-assessment of Table 1 Variables After Bonferroni Correction***

| Variable | Original P-value | Significant (P < 0.05)? | Significant (P < 0.00217)? | Conclusion after Correction |
| --- | --- | --- | --- | --- |
| Age | 0.145 | No | No | Not Significant |
| BMI | 0.390 | No | No | Not Significant |
| Gender | 0.732 | No | No | Not Significant |
| ****Hypertension**** | ****0.025**** | ****Yes**** | ****No**** | ****Not Significant**** |
| ****Diabetes Mellitus**** | ****0.001**** | ****Yes**** | ****Yes**** | ****Significant**** |
| Coronary Artery Disease | 0.356 | No | No | Not Significant |
| History of Cerebral Infarction | 0.564 | No | No | Not Significant |
| Smoking History | 0.338 | No | No | Not Significant |
| ****History of Alcohol Abuse**** | ****0.004**** | ****Yes**** | ****No**** | ****Not Significant**** |
| Mechanism of Injury | 0.485 | No | No | Not Significant |
| ****ASIA Grade (A-B vs C-D)**** | ****<0.001**** | ****Yes**** | ****Yes**** | ****Significant**** |
| ****Polytrauma**** | ****0.019**** | ****Yes**** | ****No**** | ****Not Significant**** |
| Time from Injury to Admission | 0.230 | No | No | Not Significant |
| Time from Admission to Surgery | 0.134 | No | No | Not Significant |
| ****Operative Duration**** | ****0.001**** | ****Yes**** | ****Yes**** | ****Significant**** |
| ICU Stay | 0.262 | No | No | Not Significant |
| Hospital Stay | 0.124 | No | No | Not Significant |
| Blood Loss | 0.072 | No | No | Not Significant |
| ****Intraoperative Blood Transfusion**** | ****<0.001**** | ****Yes**** | ****Yes**** | ****Significant**** |
| Preoperative Hemoglobin | 0.110 | No | No | Not Significant |
| ****Postoperative Hemoglobin**** | ****<0.001**** | ****Yes**** | ****Yes**** | ****Significant**** |
| Preoperative Albumin | 0.057 | No | No | Not Significant |
| Postoperative Albumin | 0.230 | No | No | Not Significant |

**Summary:**After the Bonferroni correction, only 5 variables remained statistically significant in Table 1: Diabetes Mellitus, ASIA Grade (A-B), Operative Duration, Intraoperative Blood Transfusion, and Postoperative Hemoglobin.

**Table 2: Multivariate Logistic Regression Analysis of Postoperative Delirium Risk in TCSCI Patients**

| Index | β | S.E. | Z | P-value | OR (95% CI) |
| --- | --- | --- | --- | --- | --- |
| ****Diabetes Mellitus**** |  |  |  |  |  |
| No | Ref. | / | / | / | 1.000 |
| Yes | 0.768 | 0.202 | 3.802 | ****<0.001**** | 2.156 (1.451-3.204) |
| ****ASIA Grade**** |  |  |  |  |  |
| C~D | Ref. | / | / | / | 1.000 |
| A~B | 1.108 | 0.235 | 4.716 | ****<0.001**** | 3.030 (1.910-4.807) |
| ****Operative Duration (per hour)**** | 0.310 | 0.091 | 3.407 | ****0.001**** | 1.363 (1.141-1.628) |
| ****Intraoperative Blood Transfusion**** |  |  |  |  |  |
| No | Ref. | / | / | / | 1.000 |
| Yes | 0.905 | 0.207 | 4.372 | ****<0.001**** | 2.473 (1.648-3.712) |
| ****Postoperative Hemoglobin (per 1 g/L decrease)**** | -0.034 | 0.008 | -4.250 | ****<0.001**** | 0.967 (0.952-0.982) |

Justification for Including "History of Alcohol Abuse": Although "History of alcohol abuse" did not survive the Bonferroni correction in the univariate analysis (P=0.004 > 0.00217), it is a well-established clinical and pathophysiological risk factor for delirium. As a sensitivity analysis, we re-introduced it into the multivariate model. It remained a statistically significant independent predictor (OR=1.929, 95% CI: 1.259–2.957, P=0.003) without causing multicollinearity (all VIFs < 5). Therefore, after careful consideration and to enhance the clinical relevance and completeness of our prediction tool, we decided to retain "History of alcohol abuse" in the final model. We have transparently explained this decision in the revised manuscript.
